# Mouse germ line mutations due to retrotransposon insertions

**DOI:** 10.1186/s13100-019-0157-4

**Published:** 2019-04-13

**Authors:** Liane Gagnier, Victoria P. Belancio, Dixie L. Mager

**Affiliations:** 1 Terry Fox Laboratory, BC Cancer and Department of Medical Genetics, University of British Columbia, V5Z1L3, Vancouver, BC Canada; 2 Department of Structural and Cellular Biology, Tulane University School of Medicine, Tulane Cancer Center, Tulane Center for Aging, New Orleans, LA 70112 USA

**Keywords:** Endogenous retroviruses, Long terminal repeats, Long interspersed elements, Short interspersed elements, Germ line mutation, Inbred mice, Insertional mutagenesis, Transcriptional interference

## Abstract

Transposable element (TE) insertions are responsible for a significant fraction of spontaneous germ line mutations reported in inbred mouse strains. This major contribution of TEs to the mutational landscape in mouse contrasts with the situation in human, where their relative contribution as germ line insertional mutagens is much lower. In this focussed review, we provide comprehensive lists of TE-induced mouse mutations, discuss the different TE types involved in these insertional mutations and elaborate on particularly interesting cases. We also discuss differences and similarities between the mutational role of TEs in mice and humans.

## Background

The mouse and human genomes harbor similar types of TEs that have been discussed in many reviews, to which we refer the reader for more in depth and general information [1–9]. In general, both human and mouse contain ancient families of DNA transposons, none currently active, which comprise 1–3% of these genomes as well as many families or groups of retrotransposons, which have caused all the TE insertional mutations in these species. As in humans [4], the mouse genome contains active retrotransposon families of long and short interspersed repeats (LINEs and SINEs) that can cause germ line mutations via new insertions but, in contrast to humans, the mouse also contains several groups of retrotranspositionally active endogenous retroviral elements (ERVs) that are responsible for most reported insertional mutations.

### ERVs/LTR retrotransposons

ERVs are the result of retroviral infections or retrotranspositions in the germline. The general structure of an ERV is analogous to that of an integrated provirus, with flanking long terminal repeats (LTRs) containing the transcriptional regulatory signals, specifically enhancer, promoter and polyadenylation motifs and often a splice donor site [10, 11]. Sequences of full-length ERVs can encode *gag, pol* and sometimes *env,* although groups of LTR retrotransposons with little or no retroviral homology also exist [6–9]. While not the subject of this review, ERV LTRs can often act as cellular enhancers or promoters, creating chimeric transcripts with genes, and have been implicated in other regulatory functions [11–13]. The mouse genome contains many different groups of ERVs and related LTR retrotransposons that together comprise ~ 10% of the sequenced genome [1] and which have been characterized to varying extents [6, 9, 14, 15]. ERVs in mouse and other vertebrates are generally categorized into three classes. Class I ERVs are most related to the exogenous gamma-retroviral genus, Class II to beta- and alpha-retroviruses and Class III to spuma-retroviruses [6, 9]. The very large non-autonomous MaLR (mammalian apparent LTR retrotransposon) group is also considered Class III but has only very small traces of retroviral homology. Different mammals have distinct collections of ERVs and the mouse is unusual in having a much higher fraction of Class II elements compared to humans or other mammals [1, 6]. For all but very young groups, the majority of ERV loci exist only as solitary LTRs, the product of recombination between the 5′ and 3′ LTRs of integrated proviral forms [16, 17]. Moreover, for ERVs that have not undergone this recombination event, most have lost coding competence due to mutational degradation over time.

**Fig. 1:**
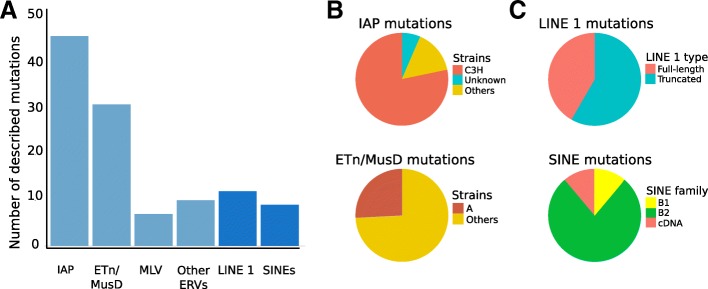
Distribution of mouse mutations caused by TE insertions. **a** Numbers of published mutations caused by different TE types. **b** Strain bias for IAP and ETn/MusD insertional mutations. **c** Upper panel – proportion of LINE1 insertional mutations that are full length or near full length. Lower panel shows high proportion of B2 SINEs among insertional mutations

Unlike human ERVs that are likely no longer capable of autonomous retrotransposition [18, 19], some mouse ERVs are retrotranspositionally active and are significant ongoing genomic mutagens in inbred strains, causing 10–12% of all published germ line mutations via new integration events [1, 20]. The large Intracisternal A-particle (IAP) ERV group is responsible for close to half the reported mutations due to new ERV insertions, with the Early Transposon (ETn)/MusD ERV group also contributing substantially [20](Fig. 1a). These groups and other mutation-causing ERVs will be discussed in more detail in the subsequent relevant sections. The majority of mutagenic ERV insertions occur in introns and disrupt normal transcript processing (e.g. splicing and polyadenylation) to varying degrees, a mechanism well recognized since the 1990s [21–25] and discussed further below.

### Long interspersed repeats (LINEs)

LINE-1s (L1s) are autonomous non-LTR elements that have accumulated to as many as 500,000 copies in both mouse and human genomes using a copy-and-paste mechanism of amplification [1–3, 26]. Full length L1s are 6–7 kb and contain two open reading frames (ORFs) encoding ORF1p and ORF2p, with the latter having endonuclease and reverse transcriptase activity [27–30]. The number of potentially active L1s (i.e. full-length elements containing intact ORFs) varies significantly between human and mouse. Bioinformatics analyses of the reference genomes have documented 2811 mouse and 146 human L1s that are fully structurally intact [31]. Functional studies have estimated numbers of active L1s to be ~ 3000 for mouse [32] and 80–100 for human [33]. In contrast to the human genome that has had a single subfamily of LINEs active at any given evolutionary time, the mouse genome contains three concurrently active L1 subfamilies (T(F), A, and G(F)) [32, 34] that are insertionally polymorphic among strains [17, 35]. One of the distinguishing features of these subfamilies is the differing 5′ monomer tandem repeats which, when combined with a downstream non-monomeric sequence, form their 5′ UTRs [36]. The 5′ UTR also contains the L1 pol II promoter, which occurs downstream of the transcriptional start site [37, 38], an arrangement common to non-LTR retrotransposons [39], allowing the promoter to be retained in the L1 mRNA.

Mouse and human L1s contain promoters, splice and polyadenylation signals in both sense and antisense directions that are utilized during L1 and host gene transcription, also sometimes leading to the formation of chimeric mRNAs [40–44]. As with ERVs [20, 45], such cis-acting sequences are a likely reason for the negative impact of some intronic L1 insertions on gene expression [43]. De novo L1 inserts can vary in size from just a few bases to those containing a full-length L1 sequence [26], with the vast majority of such inserts being 5′-truncated to varying extents. Although the exact mechanisms underlying this truncation phenomenon remain unclear, there is a positive correlation between the frequency of retrotransposition and insert length [46], and cellular DNA repair interference with L1 integration may play a role [47, 48].

Sporadically, new germ line L1 insertions cause mutations when they land in or near a gene in human [4] or mouse (discussed below), and somatic insertions can also occur, although few of the latter have been shown to exert a significant biological effect [49–51]. Mutagenic L1 inserts can potentially disrupt normal gene function or expression by interfering with it directly or by introducing deletions or complex genomic rearrangements that are sometimes associated with the integration process [3, 52]. In addition to introducing de novo insertions containing L1 sequences, L1 can mobilize flanking genomic sequences as well. This occurs as a result of their incorporation into the nascent L1 mRNA generated by either inaccurate/upstream transcriptional initiation (5′ transduction) or inefficient transcriptional termination at the L1 3′ polyadenylation site resulting in readthrough and 3′ transduction [3, 53, 54]. Recent analysis of endogenous L1 expression in human cell lines determined that only about a third of expressed L1 loci generate such readthrough transcripts [55] but a similar analysis has not been performed for mouse. The uniqueness of these transduced sequences are often useful in identifying the source L1 element responsible for a newly retrotransposed copy [56].

### Short interspersed repeats (SINEs)

SINE elements are non-autonomous retrotransposons, as they do not encode proteins involved in their amplification. As with human Alu SINE sequences [57], mouse SINEs have been shown to be retrotransposed by mouse L1 [58]. Only one of the two L1 proteins (ORF2p) is sufficient to drive Alu SINE mobilization in tissue culture [57], although ORF1p enhances the process [59]. Both mouse and human L1s can efficiently mobilize their non-orthologous SINEs, suggesting that such a symbiotic relationship has evolved multiple times [58–62]. There are several SINE classes in the mouse genome that together comprise ~ 8% of the genome [1]. Among these are B1, B2, B4/RSINE, ID, and MIR. New mutagenic insertions have been documented for B1 and B2 (see below), indicating that at least some copies are still potentially active. B1 (like human Alu) is derived from 7SL RNA, and B2 is derived from tRNA [3]. B1 and B2 SINEs are both present at very high genomic copy numbers: ~ 560,000 for B1 and ~ 350,000 for B2 [1]. Like mouse L1s and ERVs, these mouse SINEs are insertionally polymorphic in inbred strains [17, 63, 64].

### Cataloging TE-induced mouse mutations

We assembled lists of mutations caused by TEs by perusing the literature and by querying the Mouse Genome Informatics (MGI) database of mutant alleles [65]. In October 2018 we obtained lists from MGI of all spontaneous mutant alleles that listed “viral”, “transposon” or “insertion” as the cause and extracted all relevant cases through manual curation. To avoid ascertainment bias, we excluded cases where investigators were specifically screening for effects of insertionally polymorphic TEs [35, 66, 67]. While such cases can show effects on gene expression, observable phenotypes due to these insertionally polymorphic TE insertions were not reported in the aforementioned studies. In addition, we excluded cases where the insertion event likely occurred in cultured ES cells used to produce transgenic mice. Nearly all arose spontaneously but two cases of mutations occurring during a chemical mutagenesis experiment, but likely not caused by the chemical mutagen, were also included. This search resulted in a total of 115 TE insertion mutations. Ninety-four of these were caused by insertions of ERVs/LTR retroelements and 21 were L1 or L1-mediated (Fig. 1). In the case of the ERV mutations, the tables shown here are updates of previously published lists [1, 20, 68].

### IAP insertion mutations

The group of ERVs responsible for the most reported mutations are the IAP elements. IAP sequences are Class II elements and are highly abundant in the mouse [6, 69]. Different estimates for IAP copy number exist in the literature but a recent analysis of all sequences annotated “IAP” by Repeatmasker [70] found ~ 3000 solitary LTRs and ~ 2800 full-length or partial full-length elements in the reference C57BL/6 genome [71]. Of the latter, ~ 1000 have 5′ and 3′ LTRs that are 100% identical, indicative of a very young age, and most of these belong to the IAPLTR1 or 1a subtypes [71]. As expected for such a young ERV group, IAP elements are highly insertionally polymorphic among inbred mouse strains [17, 66, 67, 72]. Although ~ 200 IAP sequences (IAPE elements) contain an *env* gene [73], most do not. Loss of *env* and other specific genetic modifications facilitated adoption of an intracellular retrotranspositional life cycle by IAPs [74] resulting in their accumulation to high copy numbers as genomic “super spreaders” [75]. Besides the lack of *env*, there are a few common partly deleted proviral forms [69] with the most notable being the 1Δ1 subtype, which has a 1.9 kb deletion removing part of *gag* and *pol,* resulting in an ORF encoding a novel gag-pol fusion protein. Although retrotransposition of 1Δ1 proviruses is non-autonomous, requiring gag and pol proteins in *trans* from other IAPs [76], this subtype is responsible for the great majority of new IAP insertion mutations [20]. Interestingly, it has been shown that the gag-pol fusion protein functions in *cis* to facilitate retrotransposition [77]. Together with a generally higher level of 1Δ1 transcripts compared to full length IAP mRNAs (see below), this *cis* effect could explain why most new insertions are of the 1Δ1 subtype.

Although transgenic experiments have shown expression of an IAP LTR only in the male germ line [78], endogenous IAP transcription is also detectable in embryogenesis as early as the two cell stage and appears highest in the morula and blastocyst stages [79]. Moreover, at least some IAP elements can be transcribed in normal somatic tissues, particularly in the thymus, where a specific subtype of IAP LTR shows transcriptional activity [80, 81]. Notably, the levels of 1Δ1 5.4 kb IAP transcripts are comparable or often more abundant than full-length IAP transcripts in different tissues or cell types [69, 80, 82], although the former are present in lower copy numbers [69, 71, 83]. The molecular mechanisms underlying the generally higher transcript levels of 1Δ1 elements are unknown but one possibility is that these elements are more likely to escape the general epigenetic transcriptional repression of IAPs by DNA methylation and repressive histone modifications [84–87].

Table 1 lists mouse germ line mutations caused by insertions of IAPs. Somatic insertions of IAP elements can also occur and cause oncogene or cytokine gene activation in mouse plasmocytomas, myelomas and lymphomas [88–90], likely due to the fact that some IAP LTRs are transcriptionally active in lymphoid tissues [80, 81]. Most of the germ line insertions occur in gene introns and disrupt transcript processing, notably splicing and polyadenylation (Table 1) [20]. However, several IAP-induced mutations involve ectopic gene transcription promoted by an upstream or intronic inserted LTR that is regulated by DNA methylation [20, 91]. In these cases, the IAP is oriented in the opposite transcriptional direction with respect to the gene and it is an antisense promoter within the LTR that is responsible for the ectopic gene transcription. For a number of such cases, including the most well studied A^vy^ allele of *agouti* [92], variable establishment of epigenetic repressive marks on the IAP LTR result in variable expressivity of the mutant (IAP) allele in genetically identical mice and have been termed metastable epialleles [91, 93]. Interestingly, a recent genome-wide screen for other IAP metastable epialleles in C57BL/6 mice identified ~ 100 such loci, with an enrichment of flanking CTCF binding sites as the primary distinguishing feature [94].

**Table 1:** IAP insertions

^a^Mutation	MGI ID^b^	Strain of origin	Year mutation arose	Location & Orientation^c^	Mutational mechanism(s) or effects	References
^*a*^ *A* ^*hvy*^	1855945	C3H/HeJ	~ 1994	Exon 1C,-^c^	IAP forms 5′-end of transcript^d^, Regulation by methylation	[181]
^*a*^ *A* ^*iapy*^	1856403	C57BL/6J xC3H male	1992	Intron, −	IAP forms 5′-end of transcript^d^, Regulation by methylation	[182]
*A* ^*iy*^	1855933	C3H/HeJ	1964	Intron 1, −	IAP forms 5′-end of transcript^d^, transcript contains internal IAP sequence	[183]
^*a*^ *A* ^*vy*^	1855930	C3H/HeJ	1960	Exon 1A, −	IAP forms 5′-end of transcript^d^, Regulation by methylation	[92, 183]
*Adamts13* ^*s*^	3579136	unknown, present in several strains	?	Intron 23, +	IAP causes premature polyadenylation and protein truncation. Reduced enzyme activity but no obvious phenotype	[184, 185]
*Ap3d1* ^*mh-2J*^	1856084	C3H/HeJ	Before 1988	Intron 21, +	Transcript contains internal IAP sequence, Truncated protein	[186]
*Atcay* ^*ji-hes*^	1856898	C3H/HeJ-Tyr^c-a^	1987	Intron 1, +	Transcript contains internal IAP sequence	[187]
*Atp2b2* ^*jog*^	3664103	DSO, derived from C3H/He-Pw hybrid	2000	Intron,+	Reduced mRNA levels, likely by terminating at poly(A) in IAP, also cryptic splicing suggested	[188]
*Atrn* ^*mg*^	1856081	C3H – Swiss stock cross	1950	Intron 26, −	Transcript contains internal IAP sequence, Truncated protein, Likely termination in IAP	[189]
*Atrn* ^*mg-L*^	2156486	C3H/HeJ	1981	Intron 27, +	Transcript contains internal IAP sequence, Reduced protein levels, Likely termination in IAP	[189]
^*a*^ *Axin1* ^*Fu*^	1856035	Bussey stock	Before 1931	Intron 6, −	IAP forms 5′-end of transcript^d^, Regulation by methylation, and transcript contains internal IAP sequence, Truncated protein	[190, 191]
^*a*^ *Axin1* ^*Fu-kb*^	1856037	unknown	mid 1970s?	Exon 7, −	IAP forms 5′-end of transcript^d^, transcript contains internal IAP sequence, Internally deleted protein	[190]
*Clcc1* ^*m1J*^	5618134	C3H/HeSnJ	After 1947	Intron 2, +	Transcripts contains internal IAP sequence, Reduced normal mRNA levels	[192]
*Cryge* ^*No3*^	3713105	C3H/HeH × 102/E1 F1	Late 1990s	Exon 3, +	Reduced mRNA levels, Transcript contains internal IAP sequence, Truncated protein	[193]
*Dab1* ^*scm*^	1856801	Dc/Le (Dc arose in an obese stock outcrossed to BALB/c x C3H/He hybrid. One cross to C3H/HeJ, then inbred.)	1991	Intron, −	Transcript contains internal IAP sequence, Truncated protein	[194, 195]
*Dnmt3C* ^*IAP*^	N	C3HeB/FeJ From chemical induction exp.	~ 2014	Last intron, −	IAP provides alternate splice acceptor site, resulting in exclusion of Dnmt3C last exon, chimeric Dnmt3C-IAP mRNA	[97]
*Eya1* ^*bor*^	1857803	C3HeB/FeJ	1984	Intron 7, +	Transcript contains internal IAP sequence, Reduced mRNA levels	[25]
*Gata3* ^*jal*^	3027100	C3H/HeJ	1990s	Intron 3, −	Unknown	[196]
*Gpr179* ^*nob5*^	5431477	C3H	After 1951	Intron 1, +	Drastically reduces gene expression	[197, 198]
*Gria4* ^*spkw1*^	3580141 (QTL)	C3H/HeJ	1950–2002	Intron 15, +	Full-length protein expression is significantly reduced	[199]
*Gusb* ^*mps-2J*^	2152564	C3H/HeOuJ	mid 1990s?	Intron 8, +,	Reduced mRNA size and level, No enzyme activity detected	[200]
*Hps3* ^*coa-6J*^	1861609	C3H/HeJ	1999?	Exon 10, −	Transcript contains internal IAP sequence	[201]
*Hps1* ^*ep*^	1856712	C3HeB/FeJ	1957	3′-coding exon, −	Transcript contains internal IAP sequence, Protein contains IAP-encoded amino acids	[202]
*Hps6* ^*ru-6J*^	1856410	C3H/HeJ	1995	Unknown, +	Kidney: loss of expression, Brain: transcript contains IAP sequence	[203]
*Kcnq1* ^*vtg-2J*^	2389447	C3H/HeJCrl-Il2^tm1Hor^	~ 2000	Exon 2, −	IAP fusion transcript likely promoted by antisense LTR. IAP provides 5′ end of 31 bp fused in frame to the gene.	[204]
*LamB3* ^*IAP*^	2179716	C3H (C3Hf/R1 male bred separately from JAX for decades)	~ 1990	Intron/exon junction (5′), −	Transcript contains internal IAP sequence, No mRNA or protein expression	[205]
*Mc1r* ^*mpc59H*^	5791971	C3H.Pde6b	2010	Exon,?	Disruption of single exon gene	[206]
*Mgrn1* ^*md*^	1856070	C3H/HeJ	~ 1960	Intron 11, +	Reduced mRNA levels, aberrantly sized transcripts	[207, 208]
*Mgrn1* ^*md-2J*^	1856072	C3H/HeJ	1978–1993	Exon 12, +	Reduced mRNA levels, aberrantly sized transcripts	[207, 208]
*Mgrn1* ^*md-5J*^	1856519	C3H/HeJ	1978–1993	Intron 2, +	Not characterized	[207, 208]
*Oprm1* ^*IAP*^	N	CXBK recomb. Inbred of B6 & Balb	Before 1984	Exon 4,?	Decreased mRNA levels, increase in length of 3’UTR. Modifies response to opioids	[209]
*Pcnx2* ^*C3H/HeJ*^	6161760	C3H/HeJ	After 1950	Intron 19, +	Modifier of *Gria4* mutation, reduces Pcnx2 expression	[83]
*Pitpna* ^*vb*^	1856642	DBA/2J	Early 1960s	Intron 4, +	Transcript contains internal IAP sequence, Reduced mRNA & protein	[210]
*Pla2g6* ^*m1J*^	4412026	C3H/HeJ	mid 2000s	Intron 1,?	Reduces normal gene transcripts by ~ 90%	[211]
*Plcd3* ^*mNab*^	N	C3H mix	~ 2005	Intron 2, −	Causes truncated protein, LTR may act as antisense promoter?	[212]
*Pofut1* ^*cax*^	3719000	C3H/HeJ	mid 2000s	Intron 4,	Reduced mRNA and protein levels	[213]
*Reln* ^*rl-Alb2*^	1857345	C3H/HeJ From chemical ind. Exp.	~ 1990	Exon 36, −	Exon skipping, Reduced mRNA levels	[214]
*Slc35d3* ^*IAP*^	3802578	C3H/HeSn-Rab27a^ash^/JRos	1980s–1990s	Exon 1, −	IAP fusion transcript likely promoted by antisense LTR. IAP provides 5′ end of 18 bp fused in frame to the gene.	[215]
*Spta1* ^*sph-Dem*^	2388936	CcS3/Dem recomb. Con. strain from BALB/cHeA and STS/A	1991	Intron 10/exon 11 junction, +	Exon skipping, Reduced protein expression, Reduced α−/β-spectrin dimer and tetramer stability	[216]
*Tal1* ^*Hpt*^	1859843	C57BL/6J x C3HeB/FeJLe-a/a)F1.	1979	Intron 4, +	Promotes overexpression of exons 4 and 5, fusion transcripts found.	[217]
*Tnfrsf13c* ^*Bcmd1*^	2389403 (QTL)	A/WySnJ	Before 1991	Exon 3, +	Fusion transcripts, Loss of function mutation	[218]
*Tyr* ^*cm1OR*^	2153728	C3Hf/R1	1988	Upstream, −	IAP does not form 5′-end of transcript, Reduced mRNA expression	[219]
*Usp14* ^*ax-J*^	1855959	CBA or kreisler stock	Early 1950s	Intron 5, +	Transcript contains internal IAP sequence, Reduced mRNA levels, Truncated protein	[220]
*Wnt9b* ^*clf1*^	1856821	A	1920s	Downstream, +	Produces transcript antisense to *Wnt9b.*	[221, 222]
*Zfp69* ^*IAP*^	N	Unknown, present in C57BL/6, NZO, other strains	?	Intron 3 of Zfp69, +	IAP causes premature polyadenylation and alternate splicing. Loss of functional *Zfp69* in strains carrying the IAP is protective against diabetes.	[223]
*9630033F20Rik*	N	C3H/HeDiSnJ	~ 1985?	Exon 5, −	Deletion of exon 5, decreased gene expression. Occurs in “lew” mice with point mutation in *Vamp1* thought to be cause. IAP may contribute.	[224, 225]

^a^Variable phenotype or expression (metastable epiallele)

^b^ID number in Mouse Genome Informatics (MGI) database, “N” indicates not present in MGI

^c^- = antisense, + = sense,? = orientation unknown

^d^Ectopic expression of IAP-driven transcript

### IAP activity in C3H mice

Because high numbers of IAP mutations in C3H mice and high IAP insertional polymorphisms among C3H substrains have been noted before [20, 83], we investigated the strain of origin for all TE-induced mutations. For IAPs, the strain of origin could not be ascertained for three of the 46 cases but, of the remaining 43, a remarkable 84% (36 cases) occurred in a C3H strain or hybrid involving C3H (Table 1, Fig. 1b). This marked skew is not seen for mutations caused by any other retroelements, indicating that ascertainment bias cannot explain the high frequency of IAP-caused mutations in C3H mice. While the date of the mutation is difficult to determine in some cases, IAP retrotranspositions in C3H mice have spanned several decades, with the earliest reported cases in the 1950s and the latest in 2014 (Table 1). This indicates that the unusual IAP activity has been a characteristic of C3H strains for at least 60 years. Indeed, Frankel et al. have shown that at least 26 1Δ1 IAP insertions present in C3H/HeJ are absent from the highly related C3HeB/FeJ substrain [83], again indicative of ongoing activity of IAPs, particularly the 1Δ1 subtype, in this strain.

Although reasons for the numerous IAP insertional mutations in C3H strains are unknown, it is noteworthy that normal spleen, bone marrow and thymus from C3H/He mice have much higher levels of IAP transcripts compared to C57BL/6 and STS/A mice [95], suggesting that transcriptional deregulation may be involved. As well, IAPs are transcriptionally upregulated in radiation-induced acute myeloid leukemia in C3H/He mice, resulting in new insertions in the leukemic cells, most of which are of the 1Δ1 subtype [95, 96]. These observations, coupled with the fact that most new mutations in C3H mice involve the 1Δ1 subtype suggests that this IAP subtype is accumulating in the C3H genome at a faster rate than full length elements.

**Fig. 2:**
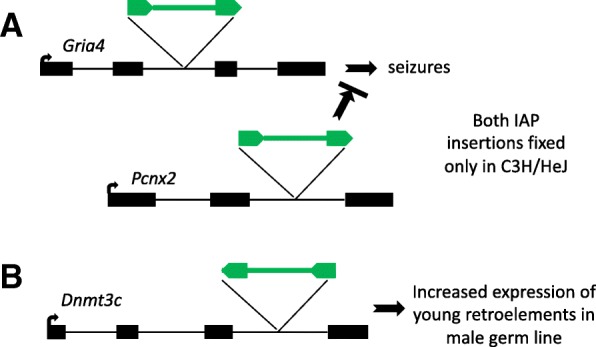
Effects of IAP insertions in C3H mice. **a** An IAP insertion in *Gria4* in C3H/HeJ causes seizures associated with spike-wave discharges but seizure episodes are much more frequent when the allele is crossed into another strain. The modifying effect in C3H/HeJ is due to another IAP insertion in *Pcnx2*, which reduces the detrimental effect of the *Gria4* mutation. **b** A new IAP insertion in the previously unknown *Dnmt3c* gene was detected in a C3HeB/FeJ colony during a screen for genes involved in retrotransposon silencing in the male germ line. See text for references. Black boxes are gene exons and green arrows and lines represent IAP LTRs and internal sequences. Numbers of exons/introns and distances are not to scale

Two recent reports illustrate the prudence of considering IAP induced mutations whenever working with C3H mice (Fig. 2). In the first case, Frankel et al. found that an IAP insertion in the *Pcnx2* gene in C3H/HeJ mice (*Pcnx2*^*C3H/HeJ*^) reduces expression of this gene, which in turn mitigates the effect of an IAP insertion in *Gria4* (*Gria4*^*spkw1*^) which causes seizures [83]. Hence one IAP insertion modifies the effect of another (Fig. 2a). In another intriguing example, Barau et al. conducted a screen in C3HeB/FeJ mice using *N*-ethyl-*N*-nitrosourea (ENU) mutagenesis to identify genes involved in retrotransposon silencing in the germ line [97]. They identified several lines with the same mutation, indicating it was not induced by ENU but rather was spontaneous. This mutation was an IAP element inserted in an intron of a gene, annotated as a non-functional pseudogene, that formed as a tandem duplication of *Dnmt3B*. Barau et al. showed that this gene, now termed *Dnmt3C*, is indeed a functional DNA methyltransferase responsible for methylating promoters of young retroelements, including L1 elements and IAPs, in the male germ line [97]. Therefore, an IAP insertion facilitated the discovery of a gene involved in its own silencing (Fig. 2b).

### C3H mouse history

The C3H strain was derived by Leonard Strong from a 1920 cross of a Bagg albino female (ancestors to the BALB/c strain) and a male from Little’s strain of “dilute browns” (ancestors to the DBA strain) [98]. One of the original female progeny of this mating developed spontaneous mammary tumors and this trait was selected for or against by subsequent inbreeding to develop the C3H strain (highly susceptible to mammary tumors) and the CBA strain (highly resistant). Mouse mammary tumor virus (MMTV), the transmissible agent responsible for the early onset mammary tumors in C3H [99, 100], was later purged from most C3H related strains by pup fostering or re-derivation. In particular, the most widely used C3H substrain C3H/HeJ was re-derived to be MMTV-free at the Jackson Laboratory (JAX) in 1999 and all C3H substrains carried at JAX have been free of MMTV since that time. Because IAP mutations have continued to occur in C3H/HeJ mice after removal of MMTV (Table 1), it is unlikely that activities of the two retroviral entities are directly related. Various substrains of C3H, including the commonly used C3H/HeJ, were derived in the late 1940s and early 1950s [101].

Interestingly, there is some evidence that C3H/HeJ has a higher spontaneous mutation rate than most other strains. A multi-year study conducted at JAX from 1963 to 1969 examined over 7 million mice derived from 28 inbred strains for spontaneous observable and heritable mutations [102]. C3H/HeJ had marginally the highest overall rate of mutations but not remarkably so [102]. However, this study also documented mutational cases of “irregular inheritance” where the trait was heritable but showed very poor penetrance. Of the 35 examples of such cases, 16 (46%) arose in C3H/HeJ, even though this strain accounted for only 9.7% of the 7 million mice in the study [102]. It is tempting to speculate that at least some of these unusual cases may involve a new IAP insertion behaving as a metastable epiallele [91, 93].

### ETn/MusD insertion mutations

After IAPs, the ETn/MusD group is responsible for the next highest number of germ line mutations, with 31 cases (Fig. 1, Table 2). ETn elements were first described as repetitive sequences expressed highly in early embryogenesis [103]. Subsequent expression analyses showed that ETns are transcribed in two windows of embryonic development. First during E3.5–7.5 in the inner cell mass and epiblast and second between E8.5–11.5 in various tissues including the neural tube, olfactory/nasal processes and limb buds [103–105]. Although ETns have LTRs, they have no coding capacity and, hence, their mode of retrotransposition was initially a mystery. Based on traces of retroviral homology in canonical ETns, we identified an ERV group, termed MusD, which is the likely progenitor of ETn [106, 107] and Ribet et al. demonstrated that coding competent MusD elements provide the machinery necessary for ETn elements to retrotranspose [108]. A subsequent phylogenetic analysis of the large betaretrovirus genus classified MusD as belonging to the Class II ERV-β7 group [14]. One analysis of copy numbers of ETn and MusD in C57BL/6 found ~ 240 ETn elements, ~ 100 MusDs and ~ 550 solitary LTRs [107], and they are highly insertionally polymorphic [17, 66, 109]. As for IAP elements, loss of the *env* gene and other genetic modifications likely resulted in genomic amplification of MusD (and ETn) elements as intracellular retrotransposons [110]. In another similarity to IAPs, most germ line mutations caused by ETn/MusD are due to insertions of the non-autonomous ETn (Table 2), in particular a specific subtype ETnII-β [20]. Of the 31 cases, only three are documented to be MusD while the rest are ETn (Table 2). The reasons for this are not clear but ETn transcripts are much more abundant than MusD transcripts in embryos and ES cells [107, 111] and there is evidence that MusD is subject to greater levels of epigenetic suppression [111, 112].

**Table 2:** ETn/MusD insertions

Mutation	MGI ID^a^	Strain of origin	Location & Orientation^#^	Mutational mechanisms or effects	References
*Adcy1* ^*brl*^	1857405	ICR	Intron 14, +^#^	Transcripts contain ETn sequence, Transcripts terminate within the ETn	[226, 227]
*Bloc1s5* ^*mu*^	1856106	Stock-t	Intron 3, +	Transcripts contain internal ETn sequence	[228]
*Cacna1f* ^*nob2*^	3605845	AXB6/PgnJ	Exon 2, +	Premature termination and alternative splicing within ETn	[229, 230]
*Cacng2* ^*stg*^	1856548	A/J	Intron 2, +	Transcripts contain ETn sequence, Probable termination within ETn	[231, 232]
*Cacng2* ^*stg-wag*^	1856386	MRL/MpJ-Fas^lpr^/J	Intron 1, +	Transcripts contain ETn sequence, Probable termination within ETn	[231, 232]
*Cacng2* ^*stg-3J*^	2155891	BALB/cJ	Intron 2, +	Transcripts contain internal ETn sequence	[231, 232]
*Clcn1* ^*adr*^	1856316	A2G	Intron 12, +	Transcripts contain ETn sequence, Termination within ETn	[233]
*Dsg4* ^*hage*^	3766998	Recom. inbred of MRL/lpr x BXSB	Intron 8, +	Aberrant splicing in ETn causes in frame additional exon of 63 amino acids	[234]
*Dysf* ^*prmd*^	3055150	A/J	Intron 4,+	ETn fixed in A/J strain. Aberrant splicing, No detectable dysferlin protein	[235]
*Edar* ^*Dl-slk*^	1856017	Stock Tyrp1^b^ Dock7^m^ Rb(4.6)2Bnr	unknown	Transcripts contain ETn sequence, Termination within ETn	[236]
*Eml1* ^*heco*^	5560734	NOR-ICR	Intron 22, +	Aberrant splicing and premature termination, transcripts contain ETn sequence	[237, 238]
*Etn3* ^*Ppd*^	3665247	CD-1	1.6 kb 3′ of *Dusp9* gene, −	Upregulates *Dusp9* in ES cells, causes phenotype in Polypodia mutant	[120]
*Fas* ^*lpr*^	1856334	MRL/Mp	Intron 2, +	Transcripts contain ETn sequence, Termination within ETn	[21, 239, 240]
*Fbxw4* ^*Dac*^	1857833	SM/Ckc	10 kb 5′ of exon 1, −	No evident difference in size or abundance of transcript, MusD* insertion	[121–123]
*Fbxw4* ^*Dac-2J*^	1857834	MRL/MpJ	Intron 5, +	Small amounts of normal transcript, MusD* insertion disrupts a conserved motif in intron	[121–123]
*Fig4* ^*plt1*^	3716838	‘mixed’ (129/Ola, C57BL/6J, C3H & SJL)	Intron 18, +	Abnormal splicing from exon18 to SA site of EtnII, very low abundance of transcript	[241]
*Fign* ^*fi*^	1856870	Random bred	Intron 2, +	Transcripts contain ETn sequence, Probable termination within ETn	[242]
*Foxn1* ^*nu-Bc*^	1856110	SELH/Bc	Intron, −	Transcripts contain internal ETn sequence	[113]
*Gli3* ^*pdn*^	1856282	Jcl:ICR	Intron 3, +	5 alternatively spliced transcripts contain ETn sequence, three terminate within ETn, two terminate as wild-type	[243]
*Hk1* ^*dea*^	2151848	A/J	Intron 4, +	Probable aberrant splicing of ETn sequences into mRNA, Decreased hexokinase activity	[244]
*Hsf4* ^*ldis1*^	3056560	RIIIS/J	Intron 9, +	ETn alters splicing resulting in a chimeric truncated protein, and causes at least 100 fold up-regulation of the transcript	[245]
*Lep* ^*ob-2J*^	1858048	SM/Ckc-Fbxw4^Dac^	Intron 1, +	Transcripts contain ETn sequence, Probable termination within ETn, Loss of leptin expression.	[23]
*Mip* ^*Cat-Fr*^	1857104	A/J	Intron 3, +	Transcripts contain ETn sequence, Termination within ETn, LTR sequence translated to protein domain	[246, 247]
*Rubie* ^*SWR-J*^	5568608	SWR/J	Intron 1, +	Aberrant splicing and premature polyadenylation of the long non-coding RNA transcript Rubie which is proposed to regulate Bmp4 expression	[248]
*Sgk3* ^*fz-ica*^	3032837	Swiss	Intron 6, +	Aberrant splicing, transcript contains ETn sequence	[249, 250]
*Sil1* ^*wz*^	3055918	Recomb. inbred CXB5/ByJ	Intron 7, +	Aberrant splicing and premature termination. Transcript contains ETn sequence	[251]
*Slc6a5* ^*m1J*^	5086232	NOD.Cg-Emv30^b^ Prkdc^scid^/Dvs	Intron 5, +	MusD* insertion, Inclusion of 183 bp of MusD sequence in mRNA, complete loss of protein	[252]
*Ttc7* ^*fsn*^	1856879	A/J	Intron 14, +	Transcripts contain internal ETn sequence	[253]
*T* ^*Wis*^	1857760	A/J	splice donor of exon 7, −	8 alternatively spliced transcripts, 4 contain ETn sequence, 1 terminates within ETn, 7 terminate as wild-type, 5 have exon skipping	[254, 255]
*Tyr* ^*c-Bc3*^	1856303	SELH/Bc	Exon 1, −	Transcripts probably contain ETn sequence, Probable termination in ETn	[113]
*Zhx2* ^*BALB/cJ*^	3623885	BALB/cJ	Intron 1, +	Transcripts contain ETn sequence, Termination within ETn	[256, 257]

^a^ID number in Mouse Genome Informatics (MGI) database, “N” indicates not present in MGI

^#^- = antisense, + = sense,? = orientation unknown

*MusD insertions are bolded. All others are ETns

ETn/MusD mutations do not show an extreme strain bias as observed for IAP insertions. However, eight mutations have occurred in “A” strain mice (Fig. 1b), such as A/J, and two in the seldom used strain SELH/Bc (Table 2) which has a high incidence of exencephaly [113, 114]. Interestingly, genomic copy number estimates in different mouse strains revealed that, while there are no detectable differences in MusD numbers, A/J, SELH/Bc and CD-1 mice have two to three times more ETnII-β elements compared to C57BL/6 [107]. Transcript levels of MusD and ETnII-β in day 7.5 embryos are also higher in SELH/Bc and CD-1 compared to C57BL/6 [107].

Nearly all of the ETn mutagenic insertions occur in gene introns, in the same transcriptional direction as the gene, and disrupt normal transcript processing through utilization of canonical or cryptic signals within the ETn, notably a specific strong splice acceptor site in the LTR, coupled with either a downstream splice donor or polyadenylation signal [20, 45]. This extreme orientation bias for mutagenic insertions is also observed for the intronic IAP insertions that do not involve IAP promoter activity (Table 1). Such an orientation skew for detrimental insertions is indeed expected, given that fixed/older ERVs have an antisense bias in genes [115, 116], presumably reflecting the fact that such insertions are less likely to be potentially deleterious and selected against.

In an attempt to mechanistically understand these orientation biases, we modeled splicing events involving intronic ERVs (using computationally predicted splice and polyadenylation motifs) and surprisingly found similar predicted frequencies of alternate splicing caused by sense or antisense ERVs [45]. However, actual splicing patterns of human mRNAs with intronic ERVs suggests that suppression of splicing within antisense-oriented ERVs occurs, possibly via steric hindrance due to annealing of sense-oriented ERV mRNAs [45]. This scenario would be analogous to gene therapy approaches where oligonucleotides that anneal to and suppress the use of mutagenic splice sites are used to redirect splicing and restore gene function [117]. Although unproven, such a mechanism could contribute to the general antisense bias for neutral/fixed ERV insertions and the opposite bias for mutagenic insertions.

Unlike for IAPs, there are no documented cases of ETn promoters causing a phenotype by driving ectopic gene expression (Table 2). This is likely due at least in part to the fact that ETn/MusD LTRs are normally only transcriptionally active in embryogenesis, responding to embryonic transcription factors [118, 119], so their promoter/enhancer activity would be silent in somatic tissues where most observable but non-lethal phenotypes manifest themselves. There is, however, at least one case where enhancer effects of an ETn insertion are likely responsible for a mutant phenotype. In this example, an ETn insertion downstream of the *Dusp9* gene upregulates this gene and also causes malformations in *Polypodia* mice, although a direct link between *Dusp9* deregulation and malformations has not been shown [120].

There is an intriguing but complex story involving two of the three documented MusD insertions [121–123]. Both of these cause the *dactylaplasia (Dac)* embryonic limb malformation phenotype by insertions within (*Fbxw4*^*Dac-2J*^) or upstream (*Fbxw4*^*Dac*^) of the *Fbxw4* gene. Both are full length MusD elements that share 99.6% identity and have occurred in different mouse strains. In the former case (*Fbxw4*^*Dac-2J*^), the intronic, sense oriented MusD severely reduces the amount of normal *Fbxw4* transcripts, likely via typical transcript processing disruption or via physical disruption of a conserved, and hence potentially regulatory, ~ 1.5 kb region within the intron [123], although neither mechanism has been formally demonstrated. In the other *Dac* mutation (*Fbxw4*^*Dac*^, also termed *Dac*^*1J*^) the MusD is inserted 10 kb upstream of the *Fbxw4* gene in antisense orientation. However, no effects on the size or abundance of *Fbxw4* transcripts are evident in mice carrying this insertion, so the mechanism by which it causes dactylaplasia remains unclear [121–123].

Interestingly, the *Dac* phenotype is modified by an unlinked polymorphic locus *mdac (*modifier of dactylaplasia) [124]. In strains homozygous for the *mdac* allele (eg. BALB/c and A/J), the dactylaplasia phenotype is observed if the mice carry either *dac* mutation. However, in strains carrying the other allele *Mdac* (eg. CBA, C3H or C57BL), the phenotypic effects of the *dac* mutations are not observed [122, 124]. Although the identity of *mdac* is still unknown, it could be a gene involved in epigenetic regulation of MusD. In *mdac/mdac* mice, the 5′ LTR of the *Dac*^*1J*^ MusD element is unmethylated and enriched in active histone marks whereas this LTR is heavily methylated and enriched in repressive histone marks in mice carrying the *Mdac* allele [122]. Moreover, ectopic MusD transcript expression is observed in embryos and limb buds of *dactylaplasia mdac/mdac* mice, but not in wildtype *mdac/mdac* mice, suggesting that the increased MusD expression is due to transcription of the *Dac*^*1J*^ MusD element itself, rather than general upregulation of MusDs in the genome [122]. The *mdac* locus has been mapped to a 9.4 Mb region between markers D13Mit310 and D13Mit113 on chromosome 13 [122, 124]. Interestingly, this region includes a cluster of KRAB-ZFP (zinc finger protein) transcription factor genes. KRAB-ZFP genes are found in multiple clusters in the genome, are rapidly evolving and highly polymorphic in mice [125, 126] and some are involved in epigenetic silencing of ERVs [126]. Hence, it is tempting to speculate that the identity of *mdac* is such a gene.

### MLV insertion mutations

The murine leukemia virus (MLV or MuLV) group is the most well characterized ERV group in the mouse and has caused seven documented spontaneous mutations (Fig. 1a,Table 3). MLV is also likely responsible for retrotransposing the non-autonomous VL30 ERV involved in the *non-agouti* mutation that will be discussed in the next section. MLVs are Class I elements, belonging to the gamma retrovirus genus, entered the mouse genome less that 1.5 million years ago and still contains infectious members [127]. MLV loci are highly insertionally polymorphic among strains [128, 129] with copy numbers of ~ 20 for xenotropic MLV and ~ 40 for polytropic MLV [9]. Ecotropic copies, i.e. those able to infect only mouse cells (and not those of other species) based on env protein recognition of a cellular receptor, are present in very few copies in various strains [127]. New germ line insertions appear to occur primarily through oocyte reinfection, rather than intracellular retrotransposition [130], which has likely kept MLV copy numbers low. Ever since it was first reported that exogenous MLV can integrate into the germ line [131], MLV and MLV-based vectors have been widely used for many applications including insertional mutagenesis screens, gene therapy and oncogene discovery [132–134].

**Table 3:** MLV Insertions

Mutation	MGI ID^a^	Strain of origin	ERV location, orientation^b^	Mutational mechanisms or effects	References
*Abcb1a* ^*mds*^	3044239	CF-1	Solitary MLV LTR, Intron 22, −	Transcripts contain viral sequence, Exon skipping, Disruption of gene function.	[258, 259]
*Aifm1* ^*Hq*^	1861097	CF-1	Intron 1, +	80% decrease in transcript and protein levels, Aberrant splicing	[260]
*Hr* ^*hr*^	1856057	HRS/J	Intron 6, +	Transcripts contain viral sequence, Probable termination within LTR	[24, 137]
*Lamc2* ^*jeb*^	3609880	129X1/SvJ	Solitary MLV LTR, intron 18, +	Aberrant splicing and premature termination, low level of normal transcripts	[261]
*Lmf1* ^*cld*^	1856820	*M. m. musculus*	Intron 7,?	Transcripts contain ERV sequence, Termination within ERV	[262]
*Myo5a* ^*d*^	1856004	Fancy mice	Intron, +	Shortened and abnormally spliced transcripts that vary among tissues	[135]
*Pde6b* ^*rd1*^	1856373	unknown	Intron 1, −	Associated with nonsense mutation in gene but effect of ERV is unclear	[263]

^a^ID number in Mouse Genome Informatics (MGI) database

^b^- = antisense, + = sense,? = orientation unknown

All of the MLV mutation-causing insertions occur in gene introns and affect normal gene transcript processing to varying degrees (Table 3). The very first ERV-induced mutation to be described, over 35 years ago, was an MLV insertion causing the *dilute* coat color mutation (*Myo5a*^*d*^) in DBA/2J mice [135]. This mutation can revert due to homologous recombination between the 5′ and 3′ LTR of the full length provirus, leaving a solitary LTR at the locus [136]. Phenotypic reversion by this mechanism also occurs for the hairless mutation (*Hr*^*hr*^), another of the first documented cases caused by an MLV insertion [137].

### Insertional mutations by other class II ERVs

In addition to the ERVs discussed above, members of five other ERV groups have caused mouse mutations (Table 4). Like the IAP and ETn/MusD groups, two of the groups, ERV-β2 and ERV-β4, belong to Class II or the betaretrovirus genus as defined by *pol* homology [14]. Both of these groups are heterogeneous and relatively low in copy number. The ERV-β2 group includes mouse mammary tumor virus (MMTV) but the ERVs responsible for the four cases of mutations belong to a different ERV-β2 cluster which has internal sequences annotated in Repbase [138] primarily as “ETnERV3” with LTRs annotated as “RLTR13A” [14]. The full ERV was not sequenced for the *Nox3*^*het*^ mutation but we presume it to be an ERV-β2 since the limited LTR sequence provided matched RLTR13A or RLTR13B [139]. For the other three ERV-β2 cases in Table 4, their full sequences have been published and they are 96–99% identical to each other with the major differences being internal deletions in the *Agtpbp1*^*pcd-2J*^ and *Prph2*^*Rd2*^ elements with respect to the longer *Etn2*^*Sd*^ ERV insertion (D. Mager, unpublished observations).

**Table 4:** Other ERV Insertions

Mutation	MGI ID^a^	Strain of Origin	ERV, Location, Orientation^b^	Mutational mechanisms or effects	References
*Agtpbp1* ^*pcd-2J*^	1856536	SM/J	ERV-β2/ETnERV3, Intron 13, +	Greatly reduced full length gene transcripts	[264]
*Etn2* ^*Sd*^	1857746	Danforth’s posterior duplication stock	ERVβ-2/ETnERV3, 12 kb upstream, +	Overexpression of Ptf1a and two neighboring genes, acts as an enhancer	[140–142]
*Nox3* ^*het*^	1856606	GL/Le	Presumed ERVβ-2/ETnERV3, Intron 12, +	Transcripts show aberrant splicing from *Nox3* into retroviral element	[139, 265]
*Prph2* ^*Rd2*^	1856523	O20/A	ERVβ-2/ETnERV3, Exon 2, −	Transcripts contain entire ERV, coding sequence disruption	[266, 267]
*Ednrb* ^*s*^	1856148	Fancy mice	ERVβ-4, Intron 1, +	Aberrant splicing and premature polyadenylation	[268]
*a*	1855937	Fancy mice	VL30, Intron 1, −; ERVβ-4 within VL30, +	Smaller gene mRNA and levels 8 fold lower than in wild-type. Aberrant splicing and premature termination within the ERVβ-4	[22, 143]
*Dock7* ^*m*^	1856946	JAX Dilute Brown	MERV-L, Exon 18, +	Frameshift and premature termination	[269, 270]
*Fgf5* ^*go-moja*^	6147688	ICR	Partial MTA MaLR, Intron 2, −	498 bp insertion is combined with 9.3 kb deletion of exon 3 and flanking sequence, no detectable transcripts	[158]
*Grm1* ^*crv4*^	3664783	BALB/c/Pas	MERV-L solitary LTR, intron 4, +	Aberrant splicing and premature termination, transcripts contain LTR sequence	[271]
*Npc1* ^*m1N*^	1857409	BALB/c	Partial MERV-L, Exon 9	824 bp partial and rearranged MERV-L combined with 703 bp deletion. Transcripts contain MERV-L sequence, decreased expression, premature truncation	[159]

^a^ID number in Mouse Genome Informatics (MGI) database

^b^- = antisense, + = sense,? = orientation unknown

The above cases highlight the continual difficulties and confusion with ERV annotation. As an example, the ERV insertion causing the allele termed “*Etn2*^*Sd*^”, where the ERV likely acts as an enhancer, was reported to be an “ETn” element [140–142]. However, as discussed above, this is misleading since “ETnERV3” is a separate entity compared to the more well-known ETn/MusD group, an important distinction but likely generally overlooked. Interestingly, when the reference C57Bl/6 genome was analyzed in 2004, less than 15 ERV loci falling into the ERV-β2 group were found and none were fully coding competent [14]. Moreover, all of the ERV-β2s discussed above also lack full open reading frames. Nonetheless, the presence of these elements at sites of new mutations in other strains suggests such strains have or had coding-competent members to provide proteins in *trans,* allowing retrotransposition of defective elements. The strains in which the ERV-β2 mutations arose (Table 4) do not share close relationships so the origin of any active autonomous copies is unknown.

The ERV-β4 group [14] has been involved in two known mutations and both occurred in old “fancy mice” (Table 4). One of these mutations (*Ednrb*^*s*^) was caused by insertion of a 5 kb non-coding competent element whose internal sequence is classified as “ERV-β4_1B-I (internal)” in Repbase [138] but half of the sequence in the middle of the element actually lacks homology to retroviruses (unpublished observations). Fifteen to 20 sequences closely related to the *Ednrb*^*s*^ element exist in the C57BL/6 reference genome and, since they contain LTRs and parts of the 5′ and 3′ internal sequences highly similar to the ERV-β4 element discussed below, it is likely that this small non-autonomous group has amplified using retroviral proteins provided by coding competent ERV-β4 elements.

**Fig. 3:**
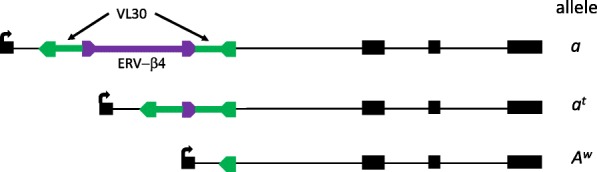
Three alleles of the *agouti* gene involving ERV insertions. The *a (non-agouti)* mutant allele is fixed in the reference strain C57BL/6. It involves a VL30 ERV and an ERV-β4 inserted within it. Partial phenotypic reversion of *non-agouti* occurs frequently. The *a*^*t*^ (black and tan) allele results from recombination between the LTRs of the ERV-β4. The *A*^*w*^ (white-bellied agouti) allele results from recombination between the VL30 LTRs. See text for references. Gene structure in black is shown to very rough scale. Green arrows and lines are the LTRs and internal VL30 sequences. Purple arrows and line depict the ERV-β4

The other mutation case involving an ERV-β4 is complex. The *a* (*non-agouti*) allele of the *agouti* gene is one of many *agouti* alleles affecting coat color [143], including four caused by IAP insertions (Table 1). The *a* allele is fixed in the reference strain C57BL/6 and is responsible for its black coat color. Molecular characterization of *non-agouti* in the early 1990s revealed that it was caused by a 5.5 kb VL30 ERV insertion in the first intron of the *agouti* gene with another reported ~ 5.5 kb segment flanked by 526 bp direct repeats found within the VL30 [22, 143]. Our perusal of the fully sequenced reference C57BL/6 genome shows that the sequence within the VL30 is ~ 9.3 kb. The mutation is reported to be caused by a VL30, which belongs to a well-studied medium repetitive non-autonomous Class I ERV group that is co-packaged with MLV, allowing its retrotransposition [144, 145]. Although VL30 is insertionally polymorphic among inbred strains [17], this is the only reported VL30-caused mutation. The nature of the insertion within the VL30 was not known at the time of analysis, but the C57BL/6 sequence shows it to be an ERV-β4 (coordinates of the full ~ 14.7 kb VL30/ERV-β4 insertion are chr2:155014951–155,029,651, GRCm38/mm10). Hence two ERV insertion events contributed to the *non-agouti* mutation, a VL30 insertion followed by insertion of an ERV-β4 within it (Fig. 3). The *non-agouti a* allele reverts at a high frequency to “black and tan” (*a*^*t*^) or white-bellied agouti (*A*^*w*^) [22, 143]. Molecular analyses by Bulman et al. showed that the *a*^*t*^ allele contains the VL30 element with a single ERV-β4 LTR and the *A*^*w*^ allele contain just one VL30 LTR [22](Fig. 3). Therefore, normal *agouti* gene expression can be partly restored by homologous recombination between the LTRs of the VL30 or the ERV-β4, as has also been observed for MLV mutations (discussed above). Notably, the ERV-β4 element involved in the *non-agouti a* allele is the only fully coding competent ERV-β4 copy in the C57BL/6 genome [14].

### Insertions by MERV-L/MaLR elements

The Class III MERV-L LTR retrotransposon group has also caused a few mutations (lower part of Table 4). MERV-L is a large, recently amplified group in the mouse with coding competent members but lacking an *env* gene [146–148]. These retrotransposons are highly expressed in the 2-cell embryo [79, 149], create viral-like particles [150] and ~ 700 full length or near full length elements exist in the reference C57BL/6 genome [148]. Therefore, the fact that there are only three reported germ line mutations caused by MERV-L insertions is somewhat paradoxical. Despite the high transcript level and particle formation by MERV-L at the two cell stage, it appears that any fully retrotranspositionally competent members are very rare or effectively blocked from completing retrotransposition by host defense mechanisms. Indeed, MERV-L elements amplified in two major bursts in mouse evolution, approximately 2 and 10 million years ago [147] and it is possible that host genetic adaptations as a result of a host-virus “arms race” [151] have effectively repressed further MERV-L expansion. Interestingly, MERV-L and associated MT MaLR LTRs have been co-opted to drive expression of genes and other transcripts involved in early embryogenesis and zygotic genome activation [79, 152–154] and there is evidence that MERV-L expression is important for embryonic development [155].

Insertion of a partial MTA MaLR element, belonging to a large young group of non-autonomous retrotransposons related to MERV-L [15, 156], and also highly expressed in early embryogenesis [153, 157], has contributed to a mutation in the *Fgf5* gene [158]. However, this case and the MERV-L insertion causing the *Npc1*^*m1N*^ mutation [159] are both partial elements and are coupled with genomic deletions, so the order of events resulting in these mutations is unclear. It is noteworthy that two of the four cases associated with Class III MERV-L/MaLR mutagenic insertions involve rearrangements of the ERV itself as well as genomic deletions. Interestingly, MaLR elements are associated with formation of independent hypervariable minisatellite sequence arrays in both human and mouse [160, 161], suggesting that these elements may foster genomic recombination and rearrangements.

### LINE1 insertion mutations

**Fig. 4:**
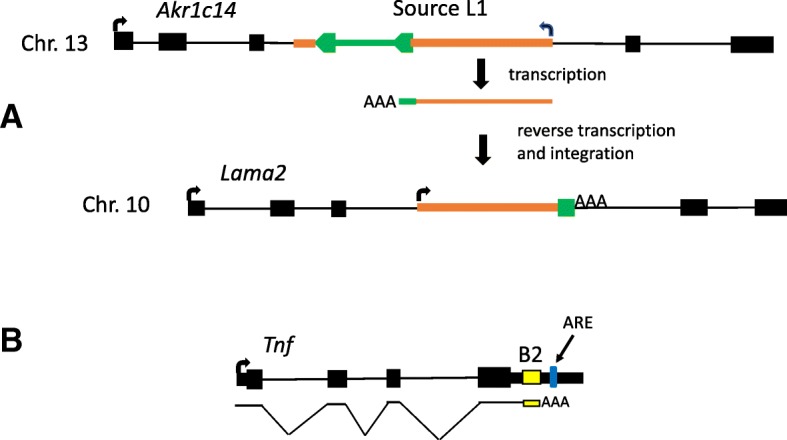
**a** Transduction of IAP LTR by an L1. A full length L1MdTf element interrupted by an IAP ERV exists in intron 3 of the *Akr1c14* gene on chromosome 13. This L1 is the source element responsible for the *Lama2*^*dy-Pas*^ mutation, with the newly inserted sequence polyadenylated in the IAP LTR. Thick orange lines are L1 genomic sequences and thin orange lines represent L1 RNA. The IAP LTRs and internal sequences are in green. Genes and number of exons are not to scale. **b** B2 insertion causing gene upregulation. The *TNF*^*BPSM1*^ mutation is a B2 insertion (in yellow) in the 3′ UTR of *Tnf*, causing *Tnf* upregulation due to polyadenylation within the B2 which removes the negative regulatory ARE (AU rich element) from the *Tnf* mRNA. Mice with this mutation have heart disease and arthritis due to overexpression of TNF. B2 is yellow and thicker black boxes are coding sequences

Our literature and MGI database search resulted in a list of 12 germ line mutations caused by L1 insertions (Table 5, Fig. 1). Of the 11 where the length and/or sequence of the insertion was published, five are full length (or nearly full length) and six are partial elements, with the shortest being only 81 bp. All five full length insertions belong to the L1MdTf family, subtypes I or II, which are among the youngest L1 subfamilies, each with over 1000 full length elements in C57BL/6 [34]. (Note that some revisions and updates to L1 subfamily classification and nomenclature have occurred [34]). In two cases, the source L1 element could be identified due to inclusion of flanking transduced sequence at the new insertion site. In the *Nr2e3rd*^*7*^ mutant allele, the L1 insertion includes 28 bp of 5′ transduced sequence, allowing the source element to be traced to the L1 at chr4:21650298–21,656,544 (GRCm38/mm10) [162]. The other case (*Lama2*^*dy-Pas*^) is interesting in that it involves an IAP LTR and an L1 [163]. While not reported as an L1 3′ transduction event in the original paper [163], our perusal of the inserted sequence (Genbank accession AJ277888) revealed that the L1 has transduced the IAP LTR, with the inserted sequence polyadenylated within the 5′ LTR (Fig. 4a). The source L1 has a 3.7 kb partly deleted IAP element inserted within it, so that ~ 700 bp of the 3′ end of the L1 occurs on the other side of the IAP (coordinates of the source L1/IAP are chr13:4065522–4,076,041, GRCm38/mm10). Another L1 insertion (*Pde6c*^*cpfl1*^), which occurred in a recombinant inbred strain established from a C57Bl/6 and BALB/c intercross, has the classical molecular structure of a 3′ transduction event [164]. However, there is no L1 element in either the sequenced C57BL/6 or BALB/c genomes at the original location of the transduced sequence (unpublished observations), which occurs in an intron of the *Diaph2* gene [164]. Therefore, the simplest explanation is that an L1 inserted in the *Diaph2* gene in the particular mouse colony being used and then retrotransposed again, creating the *Pde6c*^*cpfl1*^ allele.

**Table 5:** L1 Insertions

Mutation	MGI ID^a^	Strain of origin	L1 length, location and orientation^b^	Mutational mechanisms or effects	References
*Atp7a* ^*Mo-ca*^	2387450	unknown	81 bp L1, exon 10, −	Aberrant splicing causing in-frame loss of 10 amino acids	[272]
*Dab1* ^*yot*^	1857750	Chimera of 129 and C57BL/6	Rearranged partial L1MdTf, intron	Partial gene deletion and atypical 962 bp L1 insertion	[273]
*Frem1* ^*heb*^	1856897	AKR/J	Unknown length L1, exon 17, +	Transcript and protein truncation	[274]
*Glrb* ^*spa*^	1856363	Random-bred stock	Full length L1MdTf_I intron 6, −	Reduced expression of normal transcript and exon skipping	[275–277]
*Lama2* ^*dy-Pas*^	6220701	Non-inbred Agouti	Nearly full length L1MdTf_II and 3′ transduction, intron 34, +	Transcript contains internal IAP sequence, Truncated protein. L1-mediated retrotransposition event involving an L1 and 3′ transduced IAP LTR	[163]
*Lyst* ^*bg*^	1855968	C3H/Rl X 101/R1 (from radiation exp.)	1.1 kb 5′ truncated L1, intron, +	Two alternatively spliced transcripts containing L1 sequence results in two truncated forms of the protein	[278, 279]
*Mitf* ^*mi-bw*^	1856089	C3H	Full length L1MdTf_II, intron 3, +	Decreased expression and exon skipping of two isoforms and abolished expression of the third isoform	[280]
*Nr2e3* ^*rd7*^	1859180	77-2C2a-special- JAX	Full length L1MdTf_I with 28 bp 5′ transduction, exon 5, −	Accumulation of incompletely spliced transcripts.	[162]
*Pde6c* ^*cpfl1*^	2657247	Recom. Inbred CXB1/ByJ	1.5 kb insertion of truncated L1 & likely 3′ transduction, Intron 4, −	Aberrant splicing within inserted sequence causes inclusion of extra 116 bp exon and frame shift.	[164]
*Reln* ^*rl-Orl*^	1856416	BALB/c	Full length L1MdTf_I exon 59, +	220 bp deletion in mRNA due to skipping of exon containing L1	[281]
*Scn8a* ^*med*^	1856078	PCT	180 bp 5′ truncated L1, Exon 2, −	Skipping of exon containing L1, loss of functional protein	[282]
*Ttn* ^*mdm*^	1856953	C57BL/6J	2.4 kb L1, exon 3, +	779 bp gene deletion and L1 insertion causes exon loss or chimeric transcripts	[283]

^a^ID number in Mouse Genome Informatics (MGI) database

^b^- = antisense, + = sense,? = orientation unknown

L1 insertions have occurred in a variety of genetic backgrounds, with no evident strain bias. The mutational effects of these insertions are as expected, with intronic L1s affecting splicing and exonic cases physically disrupting the coding sequence. Interestingly of the 12 L1 cases, half occur in gene exons and half in introns (Table 5), which is more skewed toward exons compared to the ERV insertions discussed above (Tables 1-4). It is a reasonable assumption that truncated (and hence shorter) L1 insertions might be less likely to affect transcript processing if inserted in an intron. (See also discussion of SINE insertions below). Indeed, the two shortest L1 insertions of 81 and 180 bp both occur in exons (Table 5). However, two of the five full length L1s, which are similar to size to ERVs, also occur in exons.

### SINE and other LINE1-mediated insertion mutations

Members of two mouse SINE families, B1 and B2, have caused documented mutations (Table 6). Also included in this Table is a presumed L1-mediated insertion of *Cenpw* cDNA into an exon of *Poc1a* [165]. It is noteworthy that, although higher numbers of B1 elements have accumulated during mouse evolution [1], seven of the eight mutation-causing SINE insertions are B2 with no evident strain bias (Table 6, Fig. 1c). In accord with the preponderance of B2- over B1-caused mutations, retrotransposition assays in vitro showed a higher retrotransposition rate for B2 compared to B1, although the assays were conducted in human cells [58]. It is possible that B2 is currently the more active family in inbred strains, contains some members more efficient at utilizing the L1 retrotransposition machinery and/or are more transcriptionally active in the germ line. Interestingly, Dewannieux et al. [58] found that most B1 elements have a nucleotide mutation compared to Alu elements and 7SL RNA (from which both B1 and Alu were derived) and noted that this highly conserved nucleotide is critical for 7SL RNA interaction with SRP9/14 proteins [166]. As has been shown for Alu elements [167], this interaction is expected to enhance L1-mediated retrotransposition of B1. Indeed, replacement of this nucleotide in several tested B1 elements resulted in a much higher retrotransposition rate in culture [58]. Therefore, B1 elements harboring this mutation have become the most prevalent in the genome despite the fact that the mutation lowered their ability to retrotranspose. Although the evolutionary trajectory resulting in B1 prevalence is unknown, it has been suggested that, during mouse evolution, such B1 elements have been selectively retained to minimize harm to the host [58].

**Table 6:** SINEs and other L1-mediated insertions

Mutation	MGI ID^a^	Strain of origin	Insertion type, location, and orientation^b^	Mutational mechanisms or effects	References
*Atcay* ^*ji*^	1856574	Bagg albino	B1, exon 4, +	B1 insertion causes protein truncation and rare exon skipping	[187, 284]
*Comt* ^*B2i*^	4819952	Strain variant, likely origin in Lathrop stock	B2, 3′ UTR, +	Premature polyadenylation leads to shortened 3’UTR and increased protein expression. Associated with behavioral difference.	[170, 171]
*Gsdma3* ^*Dfl*^	2385837	BALB/c	B2, exon 7, +	B2 causes premature termination, transcript includes B2 sequence	[285]
*Ndufs4* ^*fky*^	4942335	Male Dnmt3L +/− x female transgenic RIP-mOVA C57BL/6	B2, exon 3, +	B2 insertion causes exon skipping and premature termination	[286]
*Nrcam* ^*m1J*^	5444298	B6.129P2-Cnr2tm1Dgen/J	B2, exon 26, −	Aberrant splicing and premature termination	[287]
*Poc1a* ^*cha*^	2135614	DBA.B6-A^hvy^/a x DBA/2J	Gene cDNA, exon 8, −	L1 mediated insertion of *Cenpw* cDNA in exon 8. Exon 8 is skipped, reading frame preserved	[165]
*Ptpn6* ^*me-B2*^	4947257	BALB/c	B2, exon 6, +	Replacement of exon 6 sequence with B2 sequence, reading frame preserved	[288]
*Slc27a4* ^*wrfr*^	2445864	(129X1/SvJ x 129S1/Sv)F1	B2, exon 3, −	Coding sequence disruption, no mRNA or protein detected	[289]
*Tnf* ^*Bpsm1*^	5795894	C57BL/6	B2, 3’UTR, +	Premature polyadenylation leads to shortened 3’UTR and gene overexpression	[172]

^a^ID number in Mouse Genome Informatics (MGI) database

^b^- = antisense, + = sense,? = orientation unknown

Unlike the ERV mutation-causing insertions, where most cases occur in introns (Tables 1-4), all such mouse SINE insertions have occurred in exons (Table 6), which represent a much smaller genomic space. The marked bias toward exonic insertions also occurs for disease-causing Alus [4]. This could simply be due to the fact that SINEs are shorter and therefore new insertions are much less likely to significantly disrupt gene expression if inserted into an intron. Indeed, although SINEs, particularly Alus, can cause alternative splicing and exonization [168], both human and mouse SINEs are relatively enriched in introns [169] and show less evidence of selection against intronic insertions compared to ERVs or L1s [68].

As is the case for mutation-causing human Alu insertions [4], most of the mouse SINE insertions directly disrupt the gene’s coding sequence, causing exon skipping, protein ablation, truncations or amino acid replacements (Table 6). However, in the *Comt*^*B2i*^ allele, which is a strain variant present in C57BL/6 and a few other strains [170, 171] and in the *Tnf*^*Bpsm1*^ mutation [172], a B2 element inserted into the 3′ UTR causes gene upregulation, which underlies the phenotype. This effect is due to a shortened 3′ UTR caused by premature polyadenylation within the B2 and a resultant replacement or disruption of negative regulatory motifs within the UTR, which has been directly shown for *Tnf*^*Bpsm1*^ [172] (Fig. 4b).

## Concluding remarks

This review has provided a comprehensive catalog and discussion of mouse mutations caused by insertions of ERVs, LINEs and SINEs. It is clear that, among these TE types, ERV insertion mutations are the most prevalent (Fig. 1a). Through an accounting of all independent spontaneous mutant alleles in mouse, it was previously estimated that ERV insertions comprise 10–12% of all published spontaneous mutations [1, 20]. Another previous report estimated that L1 insertions account for 2–3% of mouse mutations [173], suggesting a relative ratio of ERV to L1 insertion mutations of 4 to 6. Our updated numbers (94 ERV cases and 12 L1 cases) reveal a somewhat higher ratio of approximately eight. If the nine SINE insertion cases reported here are included, the ratio of ERV to “L1-mediated” insertion mutations is ~ 4.5.

Since both human and mouse have active L1s, we can attempt to compare relative L1 recent “activity” based solely on the number of documented mutations due to L1 insertions. Both bioinformatics and functional studies [31–33] suggest that the typical inbred mouse genome harbors roughly 20–30 times more retrotranspositionally competent L1s compared to human (~ 3000 versus ~ 100–150). All else being equal, one might then expect the frequency of L1 insertional mutations to be 20–30 times higher in mouse. Recent reviews on retrotransposons in human disease report 22 cases of L1 insertions causing heritable mutations/diseases [4, 174]. To put these numbers in context, it should be remembered that many more mutations have been described in human compared to mouse. The Human Gene Mutation Database [175], lists ~ 240,000 entries as of January 2019. In contrast, the MGI database [65], lists only ~ 2100 spontaneous mutant alleles as of the same date, and many of these are non-independent entries or revertant cases. While comparing such overall numbers is fraught with caveats, they are however still useful to illustrate the point that the mouse “mutational space” is vastly understudied compared to human. Hence, the relatively low number of 12 mouse L1 mutations (when compared to the number of human L1 mutations) is not unexpected but rather simply appears low when viewed against the high numbers of ERV mutations. Indeed this number is approximately in line with expectations when compared to human, given the much higher number of active L1s but much lower numbers of all characterized mutations in mouse.

In considering L1-mediated insertion mutations as a fraction of all mutations, the numbers reported here suggest a frequency of 3–5% in mouse, building on the previous L1 estimate of 2–3% [173] and including the SINE cases. There have been various estimates for the frequency of L1-mediated mutations in human, with an early estimate of 1 in 600 (0.16%) reported by Kazazian [176]. A more recent study of the spectrum of mutations in a single gene found that TE insertions caused 0.4% of all mutations in *NF-1* [177], although it is unclear if this figure can be extrapolated to all genes. In any case, these estimates suggest that the contribution of L1 activity to overall mutational burden is at least 10 fold higher in mouse.

Concerning mouse ERVs, there are several distinct ERV groups currently able to retrotranspose at least in some strains, including the low copy number and poorly characterized ERV-β2 and ERV-β4 groups [14], previously not known to be active. Unpublished transcriptome analysis indicates that expression of both these groups is readily detectable in early embryonic stages (Julie Brind’Amour and Matt Lorincz, personal communication) but little else is known about them. The fact that new insertions have been found for such low copy number ERV groups indicates they are still mutagenic in some strains and worthy of further investigation.

Another point worth emphasizing is that, although IAP ERVs are young and have accumulated to high copy numbers in inbred strains, they perhaps do not deserve the often used designation as the currently “most active” group of mouse ERVs. This is likely true only in C3H mice and, if this strain is removed from consideration, a modest seven IAP-caused mutations can be documented to have occurred in strains unrelated to C3H (Table 1,\ Fig. 1b). This number of mutations places IAP recent “activity” more on a par with the low copy number MLV and ERV-β2 groups and suggests that the genomic expansion of IAPs in most strains has largely ceased, likely due to host defense mechanisms [86, 151, 178–180] gaining the upper hand. Exclusive of the C3H strain, the ETn/MusD group accounts for the most mutagenic ERV insertions. One possible reason for the high IAP-induced mutations in C3H mice could be a slight relaxation of repression in the germ line, so it would seem prudent for investigators to consider including this strain in studies to investigate the regulation of IAPs. This extreme strain bias for IAP activity also illustrates the difficulty in attempting to compare de novo TE insertion mutation rates in the “outbred” human population with those in the artificial environment of inbred mice. Nonetheless, the primary difference between human and mouse in terms of TE-induced insertional mutations is clearly the lack of ongoing ERV activity in modern humans.

## Abbreviations

Dac: Dactylaplasia
ERV: Endogenous retrovirus
ETn: Early transposon
IAP: Intracisternal A type particle
JAX: The Jackson Laboratory
L1: LINE-1 family
LINE: Long interspersed element
LTR: Long terminal repeat
MaLR: Mammalian apparent LTR retrotransposon
MLV: Murine leukemia virus
ORF: Open reading frame
SINE: Short interspersed element
TE: Transposable element
